# Smartwatch User Interface Implementation Using CNN-Based Gesture Pattern Recognition

**DOI:** 10.3390/s18092997

**Published:** 2018-09-07

**Authors:** Min-Cheol Kwon, Geonuk Park, Sunwoong Choi

**Affiliations:** 1 Department of Secured Smart Electric Vehicle, Kookmin University, Seoul 20707, Korea; mincheol@kookmin.ac.kr; 2 School of Electrical Engineering, Kookmin University, Seoul 02707, Korea; zzaing0502@kookmin.ac.kr

**Keywords:** gesture pattern recognition, machine learning, smartwatch, Internet of things, wearable device, convolution neural network

## Abstract

In recent years, with an increase in the use of smartwatches among wearable devices, various applications for the device have been developed. However, the realization of a user interface is limited by the size and volume of the smartwatch. This study aims to propose a method to classify the user’s gestures without the need of an additional input device to improve the user interface. The smartwatch is equipped with an accelerometer, which collects the data and learns and classifies the gesture pattern using a machine learning algorithm. By incorporating the convolution neural network (CNN) model, the proposed pattern recognition system has become more accurate than the existing model. The performance analysis results show that the proposed pattern recognition system can classify 10 gesture patterns at an accuracy rate of 97.3%.

## 1. Introduction

Recently, Internet of things (IoT) technologies that add the communication functionality to ordinary objects have been developed and are widely used [1,2]. Among these IoT devices, smartwatches are one of the most familiar and widely used devices. According to the Strategy Analytics, global smartwatch shipments increased by 34% YoY (Year-over-Year) in Q1 2018 [3]. The sales and usage of smartwatches are expected to increase in the future.

Along with an increase in the usage of smartwatches, many manufacturers are offering various smartwatch applications that meet the needs of various users. Users need various input methods to control these applications. Accordingly, the user interface for utilizing smartwatch applications is crucial, and thus, the user interface should be considered while developing a smartwatch and smartwatch applications [4,5].

Currently, most smartwatches use touchscreen and voice recognition as input methods. However, these input devices offer limited control of the user interface. A smartwatch is a device worn on the wrist, and its size and volume are compact. From the viewpoint of the touchscreen, it is difficult to install a touchscreen that is sufficiently large to offer various control functions. Such a limitation causes the user to touch at the wrong place, and user with a large hand would find it more difficult to control the interface. Second, voice recognition is currently used to write messages [6] or control simple functions with the help of a voice recognition secretary (Siri [7] or Bixby [8]). However, the voice recognition is used for natural language understanding and is not suitable for controlling a specific application. It is also less accurate when there is background noise and can distract others in a cubicle or classroom environment. Since it is difficult to install sensors or additional input devices on a smartwatch due to the size limitation, an additional external input device that would allow for a communication with the smartwatch would be necessary for better control of the user interface [9].

Therefore, in this study, we aim to use an accelerometer installed on most smartwatches to recognize user gesture patterns, which are then used for the user interface. Application control methods using gesture patterns are already being used for various devices including smartphones and notebooks. For example, shaking a smartphone changes the music, and using two fingers up and down on a notebook controls the window scroll. Such control methods allow users to register convenient and often-used gesture patterns as a control command in the system, which can be used to control all the applications simply and conveniently.

We propose a method that classifies 10 gesture patterns using the convolution neural network (CNN) classification model. In the past, support vector machine (SVM) or dynamic time warping (DTW) methods have often been used. With SVM, the process by which specific features are extracted from raw data was crucial, but with CNN, raw data are used without extracting any features to prevent data loss that may occur in the feature extraction process. DTW classifies data by quantifying the similarity level between two sets of data; however, using CNN, a more accurate and refined analysis can be performed. The proposed CNN model shows a higher accuracy level than SVM or DTW by 8.1% and 1.3%, respectively.

This article is organized as follows. Section 2 describes the related research, and Section 3 shows the proposed system design. Section 4 describes the proposed gesture pattern sorter using CNN, and Section 5 evaluates the performance of the designed system. Section 6 presents the conclusion.

## 2. Related Works

This section surveys the research efforts on gesture pattern recognition systems based on the accelerometer for mobile devices. The objective of a gesture pattern recognition system is to classify the test gesture pattern set (that the user just performed) to a certain class according to the template gesture pattern set (that the user performed earlier).

Previous research can be mainly categorized into two types: the DTW based method [10] and the machine learning based method [11]. Intuitively, the DTW method measures the distance between the test gesture patterns dataset and the template gesture patterns dataset of each class and selects the class with the minimum distance [12,13,14]. Other research has used both the Hidden Markov Model (HMM) and DTW [15]. The HMM-based method is based on the probabilistic interpretation of gesture pattern samples to model the gestural temporal trajectory.

Machine learning uses techniques that give the computer an ability to learn an objective, which is based on big data, to achieve a specific goal. This is currently one of the most favored technologies and is widely used in gesture pattern recognition techniques. There are various algorithms in machine learning. Decision tree algorithm is an estimation model that uses a decision tree to connect the observed value and target value on an arbitrary item. Since its results are easy to understand and it does not need to process data, this algorithm is widely used in gesture pattern recognition [14,16]. The SVM algorithm finds the boundary that makes the largest margin between the two data. It allows for effective handling of complex data using a kennel [17]. SVM-based methods usually offer lower computational requirements at classification time, making them preferable for real-time applications on mobile devices. The gesture pattern classification accuracy depends closely on the feature vector for SVM-based methods. The SVM algorithm is extensively applied for motion gesture recognition [14,18,19]. It is used in gesture pattern recognition research where data are analyzed in the time or frequency domain or unnecessary data are separated based on a threshold [15,20]. Finally, the logistic regression algorithm is a probabilistic model that estimates the probability of the occurrence of an event using the linear connection of independent variables. Using the algorithm, six motions based on the gyroscope, accelerometer, and linear acceleration are used to improve the user interface [21].

It is important to note that we focus on the hand gesture pattern recognition system. It should be distinguished from the human activity recognition (HAR) system wherein whole body movement is considered. HAR using wearable devices has also been actively investigated for a wide range of applications, including healthcare, sports training, and daily activity recognition [22,23,24,25].

## 3. System Architecture

**Figure 1:**
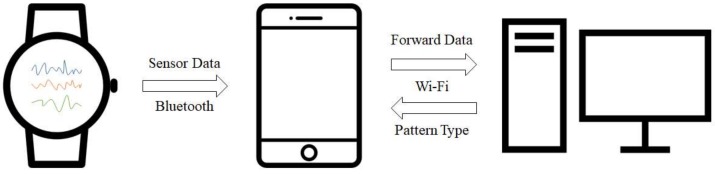
Overall system architecture.

Figure 1 illustrates the overall architecture of our system, detailing the interdependencies between each piece of equipment. Our system consists of a smartwatch, smartphone, and server. The user runs a developed application on the smartwatch to initiate the system operation. More specifically, data is periodically collected and transmitted to the smartphone using Bluetooth. Once the smartphone collects the necessary number of samples, it transmits them to the server, which has a classifier to recognize the respective gesture pattern.

### 3.1. Smartwatch

Amid the recent popularity of wrist-worn wearable devices like smartwatches or smart bands, many people use wearable devices. Most of them are equipped with an accelerometer. In this study, the Apple Watch Series 1, among the commercialized smartwatches, is used for our experiment. The Apple Watch Series 1 is equipped with a three-axis accelerometer and a Bluetooth communication module so it is very suitable for the test conducted in this study. The Apple Watch is operated using an iOS-based watchOS which allows for the development of applications through an SDK offered by Apple.

**Figure 2:**
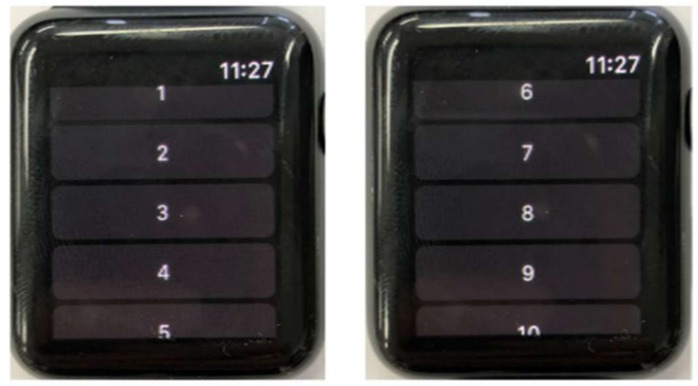
Label selection screen of smartwatch application.

A smartwatch application is developed to collect data values of the acceleration on the *x*, *y*, and *z*-axes. Moreover, while performing a data capture, the user chooses a gesture pattern label from a list of available gesture patterns on the application before starting the gesture, as shown in Figure 2. This ensures that the sensing data are classified accordingly and that these labels are used for training the classifiers. Then, during a data capture operated by the start/stop button on the application interface, sensor data from the smartwatch are collected simultaneously and sent to a smartphone. After collecting the data of acceleration and gesture pattern label, the smartwatch transmits data to a smartphone using Bluetooth communication. In this study, data are sampled from the smartwatch’s three-axis accelerometer at 10 Hz.

### 3.2. Smartphone

The smartphone application collects data from the smartwatch and transmits them to the server, which shows results classified by gesture patterns based on the received data. The smartphone is connected to the smartwatch via Bluetooth and to the server via Wi-Fi. In this study, the Apple iPhone 6 is used because it offers good connectivity with Apple Watch.

### 3.3. Server

Compared with workstations, mobile computing platforms have limited processing power; hence, it is difficult to train a machine learning model using the processing power of a mobile device. To overcome these limitations, we have adopted a cloud system that uses a server for computationally-intensive tasks. The server plays two vital roles: training the classifier and classification. The classifiers that run on the server need to be trained by the collected dataset. The server uses the trained classifiers to recognize the gesture pattern using the transmitted data from the smartphone and sends the result, i.e., one of the 10 gestures predicted by the classification model, back to the smartwatch and smartphone. The server, running the CentOS, is equipped with Intel Xeon E5-2630 2.2 GHz CPU 2EA, 256 GB RAM, and GTX1080Ti GPU 4EA. The deep learning experiment in this study was implemented using Tensorflow [26] and Keras [27].

## 4. Gesture Pattern Recognition with CNN

### 4.1. Gesture Patterns

**Figure 3:**
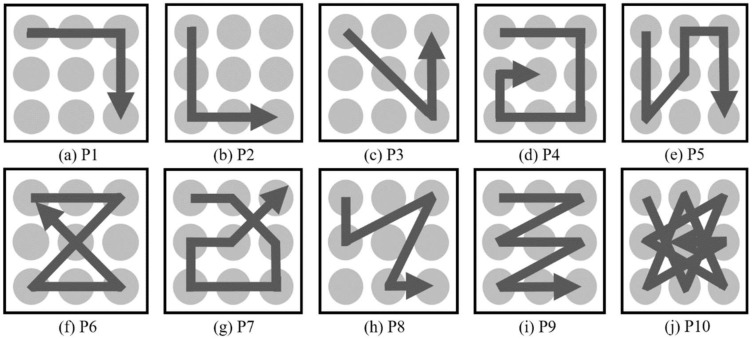
Gesture patterns.

In this study, we select and classify 10 patterns that can be used for the user interface design, as shown in Figure 3. On the two-dimension space, various patterns involving 9 dots are considered to determine 10 patterns.

**Figure 4:**
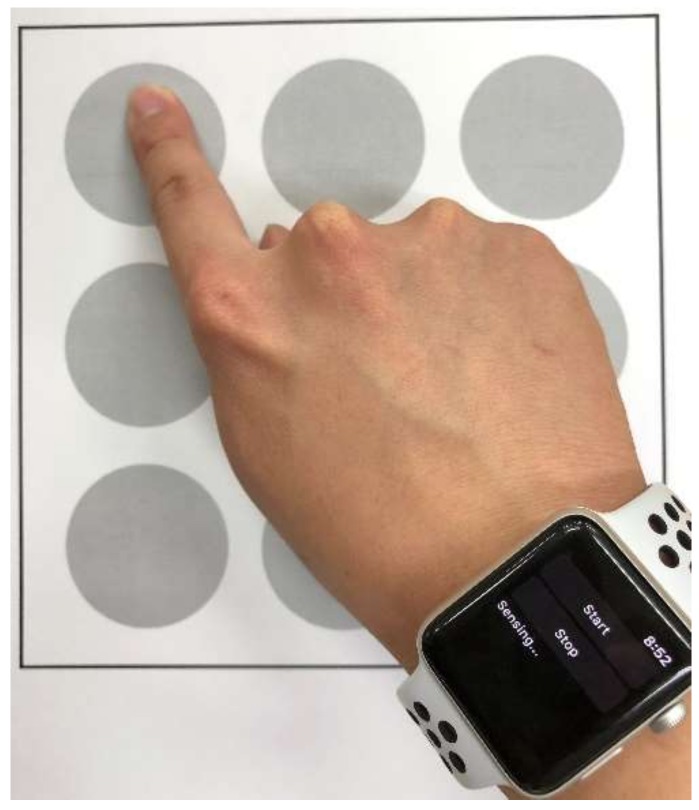
How to collect the gesture pattern dataset.

The method used to collect gesture patterns is shown in Figure 4. The participants in the experiment wear a smartwatch on the wrist that they mainly use and perform gestures from the given set of 10 gesture patterns, as shown in the Figure 3. The nine reference points in Figure 4 are offered for the participants’ convenience and the 10 gesture patterns in Figure 3 are shown to the participants who then perform each gesture with the posture, hand shape, and motion that they want to take. In this paper, the gesture patterns are performed on a 2-dimensional flat surface like a desk or a flat plate.

### 4.2. Observation on Gesture Traces

A gesture trace is described as a time series of the acceleration measurements. Let $A_{x}$, $A_{y}$, and $A_{z}$ be vectors of *x*, *y*, and *z* acceleration components according to the smartwatch axes. Let $a_{i}^{t_{j}}\;$be the value measured from the accelerometer on the *i*-axis, and in time, $t_{j}\;$, which is the *j*-th sampling time. Let n be the number of samples within the whole gesture. Therefore, we have

$$A_{x}=\left(a_{x}^{t_{1}},\;\ldots ,\;a_{x}^{t_{n}}\right),$$

$$A_{y}=\left(a_{y}^{t_{1}},\;\ldots ,\;a_{y}^{t_{n}}\right),$$

$$A_{z}=\left(a_{z}^{t_{1}},\;\ldots ,\;a_{z}^{t_{n}}\right).$$

**Figure 5:**
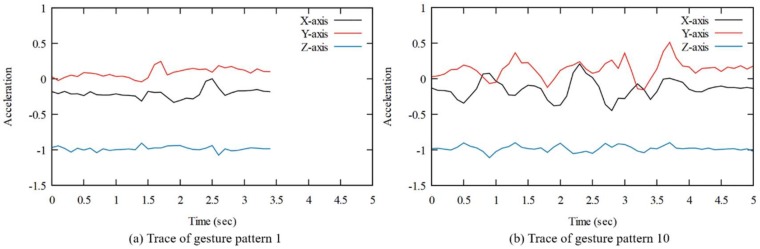
The graph of gesture 1 and 10 traces.

The raw acceleration of the gesture pattern information collected using the smartwatch consists of $A_{x}$, $A_{y}$, and $A_{z}$. Figure 5 shows the one of raw acceleration series of gesture Patterns 1 and 10. It can be seen that due to the different pattern scenarios based on the type of gesture, the amount of runtime and acceleration values vary. For example, there were two rapid fluctuations in Pattern 1, whereas there were more than five fluctuations in Pattern 10. The difference in execution time between two gestures is about 1.5 s.

### 4.3. CNN

In this section, the proposed classification model for the 10 gesture patterns is explained. The CNN algorithm is used for classification. To use the CNN algorithm, the input data size would need to be fixed. The input data size is fixed to 10 s. The collected pattern data set is 3 to 6 s long. Sampled at 10 Hz, the data at each axis has data from 30 to 60, each of which was set to 10 s long, that is, 100 in size. Thus, the total input data size is 1 × 100 × 3 where each of the *x*, *y* and *z*-axis has 100 data. Insufficient data are padded with the value at the end. For example, the trace of Figure 5b is 5 s long and then blank data from 5 to 10 s are set to the value at 5 s.

**Figure 6:**
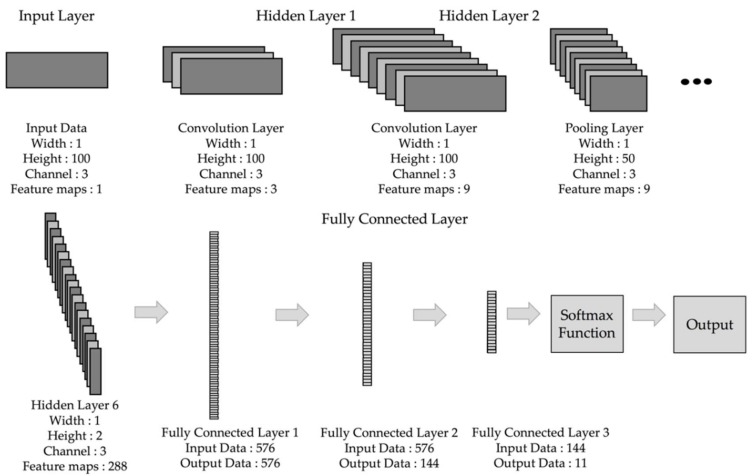
The overall architecture of the convolution neural network (CNN) used in the proposed algorithm.

Figure 6 shows the procedure of the designed CNN model. The machine learning classification model generally consists of the input, hidden, and output layers. The designed CNN model can be divided into the input, convolution, pooling, fully-connected, and output layers. The input layer enters the data to be classified based on the input format. The hidden layer consists of the convolution, pooling, and the fully-connected layers. The convolution layer is connected to some area of the input image and calculates the dot product between this connected area and its own weighted value. The pooling layer performs downsampling per dimension and outputs the decreased volume. In the fully-connected layer, all nodes are interconnected, and the result of each node is calculated by the matrix multiplication of the weight and adding a bias to it. In the output layer, all classes are converted into a probability via the Softmax function and are classified by the highest probability.

The detailed structure of the designed CNN model is shown in Table 1. Conv, Pool, and FC are the acronyms of convolution, pooling, and the fully-connected layers, respectively. It also shows the size of the patch and stride used by each layer, the output size, and the number of parameters. The convolution layer starts its feature maps from nine and increases it by two times each. The pooling layer reduces the data size by half, at which point it uses the Max pooling method, which reduces the size of data, by selecting the largest value within the size of the filter. The fully-connected layer occurs three times in total, and through the two dropouts, it reduces overfitting. The dropout rates are set to 0.5.

To learn the CNN model, the study used the Rectified Linear Unit (ReLU) [28] as an activation function, cross-entropy as a cost function, and Adam [29] as an optimizer. Training minimizes the value of loss function with respect to parameters such as weights and biases using the optimizer and the parameters are updated repeatedly. The Adam optimizer is used since it is known to achieve good results fast [29]. The learning rate is set to 0.0001.

## 5. Performance Evaluation

In this chapter, the gesture pattern recognition system designed in the previous chapter will be evaluated. The dataset used in the gesture pattern recognition system and performance evaluation index is explained, and the performance of the proposed model will be compared and discussed with the previous models.

### 5.1. Experiment Dataset

For the performance evaluation of the model, a total of 5000 experiment datasets were collected by making the ten participants perform every gesture 50 times for three days. Based on this dataset, the proposed gesture pattern recognition system was trained and tested. The training and testing process used a 5-fold validation algorithm to improve the reliability of the performance evaluation result. The number of the dataset collected by each gesture pattern is shown in Table 2.

### 5.2. Performance Measures

For the effective performance evaluation of the proposed system, we used the following four indicators: accuracy, precision, recall, and F1-score [30]. Equations (1)–(4) and Table 3 show how accuracy, precision, recall, and F1-score are derived, respectively. These four expressions are the most frequently used performance indicators for machine learning models.

(1) $$\mathrm{Accuracy}\;=\frac{\left(TP+TN\right)}{\left(TP+TN+FP+FN\right)},$$

(2) $$\mathrm{Precision}\;=\frac{\left(TP\right)}{\left(TP+FP\right)},$$

(3) $$\mathrm{Recall}\;=\frac{\left(TP\right)}{\left(TP+FN\right)},$$

(4) $$F1-\mathrm{Score}\;=2\times \frac{\left(\mathrm{Precision}\times \mathrm{Recall}\right)}{\left(\mathrm{Precision}+\mathrm{Recall}\right)}.$$

### 5.3. Proposed Model Evaluation

The performance evaluation results of the proposed gesture pattern recognition system are shown in Table 4.

The detailed results of the proposed classification model are as follows: all four performance indices are very high at about 97%. In Table 5, P1, P6, P9, and P10 show an extremely high accuracy rate of 99%, classifying almost all gestures. The classification performance of these four patterns is excellent. P7 and P8 show an estimation rate of 97%, and P3 shows a lower estimation rate of 92.2%. The detailed explanation of the confusion matrix shows that P2 and P3, and P5 and P8 have similar characteristics; therefore, we may get confused between them. Furthermore, P1 was confused with P2, P2 and P3 with P9, P4 with P3 or P9, P5 with P6 or P8, and P7 with P4, P9 or P10. Generally, all patterns show an equally high estimation rate.

### 5.4. Comparison with Other Classification Algorithms

In this section, comparison of the performance evaluation results between the proposed classification model and other classification algorithms, as discussed in Section 2, is made. In this performance evaluation, the classification model uses the SVM [17] and DTW [10] algorithms, which have been used in most of the previous studies.

One of the most important problems of the SVM algorithm is the identification of the features that will represent the data. In this study, we used 34 features which were selected from a previous paper [15]. They include time domain features (gesture time length, zero-crossing rate, mean, standard deviation, maximal, and minimal), frequency domain features (period and energy), and singular value decomposition (SVD) features (first and second column vector of the rotation matrix and relative values of the singular values). An optimal tuning of hyperparameters in SVM-based classification model is also key to achieving high classification accuracies. To obtain the lowest error rate, we performed a grid-search over the regularization parameter (C) between 1 and 10,000 and the gamma parameter (γ) between 0.0001 and 1. The radial basis function (RBF) has been used as the kernel of the SVM-based classification model.

The DTW method measures the distance between the test gesture patterns dataset and the template gesture patterns dataset of each class and selects the class with the minimum distance. In this study, the DTW-based classification model uses the nearest neighbor algorithm after obtaining DTW values between test data and all data in the training dataset.

**Figure 7:**
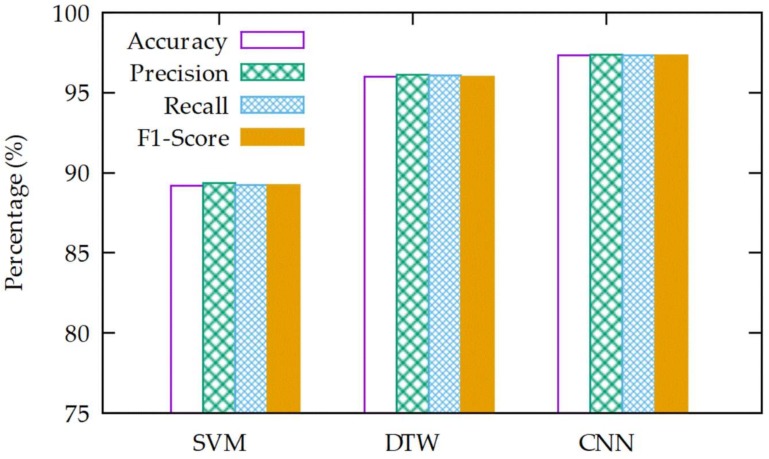
The results of the various algorithms.

The graphs representing the performance of each classification model is shown in Figure 7. Overall, the performance of the CNN-based classification model we proposed in this article is superior in comparison with other classification models, and the SVM-based classification model shows the poorest performance. On the contrary, in comparison with the SVM-based classification model, the CNN-based classification model shows an increase in accuracy of 8.1%, precision of 8.0%, recall of 8.1%, and F1-score of 8.1%; in comparison with the DTW-based classification model, it also shows an increase in accuracy of 1.3%, precision of 1.3%, recall of 1.3%, and F1-score of 1.3% [15]. In comparison with the other classification models, the CNN-based classification model shows superior performance in all performance indices. The difference in accuracy, precision, recall, and F1-score between the SVM-based and DTW-based classification models is 6.8%, 6.7%, 6.8%, and 6.8%, respectively.

### 5.5. Discussion

The classification model proposed in this paper classifies 10 gesture patterns at an average accuracy of 97.3%. Before acquiring the study results, it is assumed that the classification would cause some confusion since the classification model would detect and classify the characteristics of patterns through the accelerometer on a smartwatch. However, our results refute this assumption. For example, it is assumed that P1 and P2 would be confused as their gesture patterns are simple and the movement mechanisms are similar to each other; however, the classification model did not confuse the two patterns. Similarly, it is assumed that P10 would not be confused at all owing to its complex pattern. However, the results show that it is confused with P9 instead. While the results are different from the estimation, it was verified that the proposed classification model classified each pattern at a very high estimation rate. Furthermore, in comparison with the classification models used in the existing studies, the proposed model shows superior performance. Of course, it is noteworthy that the proposed algorithm requires more sample data to achieve good performance compared to the SVM and DTW algorithms, since it is based on a deep neural network model.

## 6. Conclusions

Owing to the recent advancements in the field of wearable device technology and an increase in its usage, there is a growing need for comfortable user interface design. However, due to various limitations of wearable devices, such as the size and volume, it is difficult to install additional input devices or sensors. Accordingly, many studies have been conducted to realize a precise and detailed user interface using various sensors that are already installed in wearable devices to track and classify user movements. Existing studies have classified simple patterns using the DTW algorithm or relatively light machine learning algorithms. However, to prove a more diverse range of functions and improve the user interface, it is necessary to increase the number of types of gesture patterns that can be recognized and improve the accuracy of gesture pattern recognition.

In this paper, the proposed classification model was used to classify 10 gesture patterns with an accuracy of 97.3%, and due to the nature of the deep-learning technology, the more data able to be accumulated, the better the accuracy will be. Not only will the advancement of such technology help realize various user interface designs, but it will also make it possible to realize user interfaces that reflect the users’ individual personality. Furthermore, it will allow for the expansion of various other functionalities and utilities, such as signature, personal security, or user identification, among others, because it enables lightweight authentication based on the physical manipulation of the device. It is believed that more diverse services will be offered based on the proposed classification model [13,31,32]. To this end, future researches may focus on gesture pattern recognition with an increased number of reference points or three-dimensional gestures.

## Figures and Tables

**Table 1:** The detailed structure of the CNN used in the proposed algorithm.

Layers	Patch Size/Stride	Number of Parameters	Output Size
Input	-	-	1 × 100 × 3
Conv1	1 × 3 × 3 × 9 + 9/1	90	1 × 100 × 9
Pool1	1 × 2/2	-	1 × 50 × 9
Conv2	1 × 3 × 9 × 18 + 18/1	504	1 × 50 × 18
Pool2	1 × 2/2	-	1 × 25 × 18
Conv3	1 × 3 × 18 × 36 + 36/1	1980	1 × 25 × 36
Pool3	1 × 2/2	-	1 × 13 × 36
Conv4	1 × 3 × 36 × 72 + 72/1	7848	1 × 13 × 72
Pool4	1 × 2/2	-	1 × 7 × 72
Conv5	1 × 3 × 72 × 144 + 144/1	31,248	1 × 7 × 144
Pool5	1 × 2/2	-	1 × 4 × 144
Conv6	1 × 3 × 144 × 288 + 288/1	124,704	1 × 4 × 288
Pool6	1 × 2/2	-	1 × 2 × 288
FC1	576 × 576 + 576	332,352	576
Dropout	-	-	-
FC2	576 × 144 + 144	83,088	144
Dropout	-	-	-
FC3	144 × 10 + 10	1450	10
Softmax	-	-	10

**Table 2:** The number of a dataset according to gesture pattern types.

Gesture Pattern	Number of Datasets
P1	500
P2	500
P3	500
P4	500
P5	500
P6	500
P7	500
P8	500
P9	500
P10	500
Total	5000

**Table 3:** Confusion matrix of two-class classification.

		True Condition	
	Total Population	Condition Positive	Condition Negative
**Predicted Condition**	Predicted condition positive	True Positive (TP)	False Positive (FP)
	Predicted condition negative	False Negative (FN)	True Negative (TN)

**Table 4:** The score of the proposed classification model.

Accuracy	Precision	Recall	F1-Score
97.3%	97.36%	97.32%	97.32%

**Table 5:** The confusion matrix of the proposed classification model.

Actual Class	Predicted Class									
	P1	P2	P3	P4	P5	P6	P7	P8	P9	P10
P1	99.1%	0.9%	0.0%	0.0%	0.0%	0.0%	0.0%	0.0%	0.0%	0.0%
P2	0.0%	96.3%	2.8%	0.0%	0.0%	0.0%	0.0%	0.0%	0.9%	0.0%
P3	0.0%	4.9%	92.2%	0.0%	0.0%	1.0%	0.0%	0.0%	1.9%	0.0%
P4	0.0%	0.0%	1.0%	98.0%	0.0%	0.0%	0.0%	0.0%	1.0%	0.0%
P5	0.0%	0.0%	0.0%	0.0%	96.7%	1.1%	0.0%	2.2%	0.0%	0.0%
P6	0.0%	0.0%	0.0%	0.0%	0.0%	99.0%	0.0%	0.0%	1.0%	0.0%
P7	0.0%	0.0%	0.0%	1.0%	0.0%	0.0%	97.1%	0.0%	1.0%	1.0%
P8	1.0%	0.0%	0.0%	0.0%	1.0%	1.0%	0.0%	96.9%	0.0%	0.0%
P9	0.0%	1.0%	0.0%	0.0%	0.0%	0.0%	0.0%	0.0%	99.0%	0.0%
P10	0.0%	0.0%	0.0%	0.0%	0.0%	0.0%	0.0%	0.0%	1.1%	98.9%
